# Thalamic Deep Brain Stimulation for Dystonic Head Tremor: A Long‐Term Study of 18 Patients

**DOI:** 10.1002/mdc3.70266

**Published:** 2025-08-27

**Authors:** Johanna M. Nagel, Marc E. Wolf, Christian Blahak, Joachim Runge, Christoph Schrader, Dirk Dressler, Assel Saryyeva, Joachim K. Krauss

**Affiliations:** ^1^ Department of Neurosurgery Hannover Medical School Hannover Germany; ^2^ Department of Neurology Universitätsmedizin Mannheim, University of Heidelberg Mannheim Germany; ^3^ Department of Neurology Katharinenhospital Stuttgart Germany; ^4^ Department of Neurology, Ortenau Klinikum Lahr Lahr Germany; ^5^ Department of Neurology Hannover Medical School Hannover Germany; ^6^ Center of Systems Neuroscience Hannover Germany

**Keywords:** deep brain stimulation, dystonia, head tremor, cervical dystonia, ventral intermediate nucleus, thalamus

## Abstract

**Background:**

Dystonic head tremor is a particular manifestation of dystonia, which is difficult to treat. Although deep brain stimulation (DBS) of the globus pallidus internus has been established as a treatment for different phenotypes of dystonia, its role in dystonic tremor has been debated. Although thalamic targets have been used for treatment of dystonic tremor of the extremities, there is limited experience with dystonic head tremor.

**Objectives:**

Here, we present our experience with thalamic ventral intermediate (Vim) nucleus DBS in a consecutive series of patients providing long‐term follow‐up.

**Methods:**

Eighteen patients with dystonic head tremor as the leading symptom underwent the implantation of quadripolar DBS electrodes into the thalamic Vim. Patients' symptoms were evaluated with the Burke‐Fahn‐Marsden Dystonia Rating Scale motor (BFMDRS‐M) and disability (BFMDRS‐D) scores, and a modified Fahn‐Tolosa‐Marin Tremor Rating Scale (mFTMTRS), preoperatively, at short‐term (3 months–2 years), and at long‐term follow‐up (>2 years).

**Results:**

There was improvement in both tremor and dystonia rating scales. Tremor scores were reduced from a value of 7.94 preoperatively to 2.0 at short term (*P* < 0.001) and to 1.71 at long‐term follow‐up (*P* < 0.001). BFMDRS‐M scores were reduced from 14.56 to 7.8 (*P* < 0.001) and to 7.0 (*P* < 0.001), and BFMDRS‐D scores from 3.88 to 2.65 (*P* < 0.05) and to 2.71 (*P* < 0.05), respectively, during chronic stimulation.

**Conclusions:**

Our results indicate that Vim DBS is a safe and efficient treatment option for dystonic head tremor with stable long‐term results. We suggest Vim DBS as an alternative to pallidal DBS in cases where head tremor is the leading symptom.

The prevalence of tremor in dystonia patients is unclear, and numbers range between 11% and 87%^1^ in the literature. Dystonic tremor has been defined as tremor that develops in a body region affected by dystonia.^2^, ^7^ Most manifestations of tremor appear in patients with cervical dystonia.^3^ In a cohort of 473 dystonia patients, Erro et al. observed tremor in 55.4%, with head tremor being the most common form (41.4% of tremor patients).^4^ Dystonic head tremor is a rare but strongly stigmatizing, irritating, and sometimes painful movement disorder.^5^, ^6^ The tremor is often jerky and aggravated when the head is turned to the opposite side of the dystonic muscle contraction.^1^ Its underlying pathophysiology is poorly understood.^1^, ^7^

The first‐line treatment of dystonic head tremor consists of botulinum toxin (BTX) injections into the involved cervical muscles. Although medication with anticholinergics (eg, trihexyphenidyl), propranolol, clonazepam, or tetrabenazine can be tried, it often shows less effect than BTX treatment.^5^ When tremor is not sufficiently responsive to BTX or medical treatment, deep brain stimulation (DBS) has been suggested as a possible alternative.^5^

DBS has become a widely accepted treatment option for several manifestations of dystonia, including focal, segmental, and generalized dystonia in particular in certain inherited and idiopathic forms refractory to medication.^8^, ^9^ Since we have introduced globus pallidus internus (GPi) DBS for the treatment of cervical dystonia in the late 1990s, the GPi has remained the main target for dystonia DBS, whereas other targets such as the subthalamic nucleus (STN) or the thalamus have been less explored.^10^, ^11^, ^12^ The effect of pallidal stimulation on dystonic tremor, however, needs further clarification, and it has been suggested to explore alternative targets, in particular, the thalamus or to use combined stimulation approaches.^12^, ^13^, ^14^, ^15^, ^16^, ^17^, ^18^, ^19^

There is only limited experience with DBS for treatment of disabling dystonic head tremor refractory to conservative treatment.^13^, ^14^, ^17^ Both DBS of the GPi and of thalamic targets, alone or in combination, have been reported in case reports and small case series.^14^, ^15^, ^16^, ^17^, ^18^, ^19^, ^20^, ^21^, ^22^, ^23^, ^24^ Of these only few focused specifically on dystonic head tremor.^15^, ^21^, ^23^, ^24^, ^25^

Although there is agreement that the best target for treatment of essential tremor and other tremor syndromes is the nucleus ventralis intermedius (Vim) or the adjacent subthalamic area,^26^ there is no consensus about the thalamic target in tremor associated with dystonia.^16^, ^23^, ^25^, ^27^ Notably, the thalamus was the main target for treatment of cervical dystonia prior to the introduction of DBS,^27^, ^28^, ^29^ and unilateral intermittent low‐frequency stimulation with electrodes implanted in the thalamus/subthalamic area was described as early as in 1977^30^ but was then discontinued because of technical problems. We have started to use bilateral Vim DBS for dystonic head tremor as early as 2000.^13^ Here we report the results of an observational study with prolonged long‐term follow‐up on a consecutive series of 18 patients.

## Methods

Here we report an observational study with retrospective analysis of data. The ratings were made directly before and after the implantation of electrodes and during the consecutive follow‐up visits. Patients with mild to moderate cervical dystonia and dystonic head tremor were referred for operative treatment. A diagnosis of dystonic head tremor was made, when head tremor and dystonic posturing of the neck were present concomitantly. Tremor was irregular, appeared with different amplitudes, and patients used sensory tricks to suppress the tremor. Cases to be included in this analysis were chosen retrospectively to evaluate only patients with dystonic head tremor, in whom tremor was the leading symptom, and who had insufficient or no response to medication, including BTX injections. We hypothesized that thalamic stimulation would improve tremor as well as dystonia in this specific patient cohort.

Patients underwent bilateral stereotactic CT‐guided implantation of quadripolar electrodes (Model 3387, Medtronic, Minneapolis, Minnesota, USA) into the thalamic Vim under local anesthesia (when possible). Stereotactic coordinates were *x* = 12–13 mm, *y* = −4 mm, *z* = 0 mm with regard to the midpoint of the intercommissural line. In 8 early patients, additional electrodes were implanted into the posteroventral lateral GPi, assisted by intraoperative microelectrode recording.^12^, ^32^ Electrodes were connected to an implantable pulse generator (IPG), (Soletra, Activa RC, or Activa PC; Medtronic), placed in the subclavicular subcutaneous/subfascial tissue. Postoperative stereotactic CT‐scans were obtained to confirm electrode placement and to exclude perioperative complications (eg, bleeding, stroke). The surgical techniques have been described in detail elsewhere.^33^, ^34^, ^35^ All operations were supervised by the senior author (J.K.K.) and carried out at Hannover Medical School or the University Medical Centre Mannheim.

In patients with combined implantation of electrodes in the Vim and the GPi, electrodes were externalized for a brief trial period of 2 to 5 days. After the trial period, only thalamic electrodes were connected to the IPG in 6 patients, whereas 2 patients had all 4 electrodes (thalamic and pallidal) connected to IPGs. In patients with thalamic DBS only, the IPG was implanted directly after the postoperative stereotactic CT was obtained. Patients received an antibiotic regime prior to skin incision and for 48 h after the operation and during the externalization phase to prevent infection.^34^

The study was conducted in accordance with the Declaration of Helsinki, and all patients provided written informed consent to use anonymized patient data for research purposes.

All patients were evaluated preoperatively, at short‐term (3 months to 2 years) and at long‐term follow‐up, using the Burke‐Fahn‐Marsden Dystonia Rating Scale motor (BFMDRS‐M) and disability (BFMDRS‐D) scores to assess the degree of dystonia, and a modified Fahn‐Tolosa‐Marin Tremor Rating Scale (mFTMTRS) to assess tremor severity (see Pauls et al. 2014^17^). The mFTMTRS included the following items: head tremor amplitude at rest, head tremor amplitude during activity, and duration of head tremor. One patient was lost to long‐term follow‐up after 13 months. All follow‐up assessments were performed after adjustment of stimulation settings. Evaluation was performed by 2 movement disorder neurologists (MEW, CB), and in case of discrepancies, assessments were reviewed on the basis of video recordings by the first and last authors (JKK, JMN).

Statistical analysis was performed with SPSS version 28 for Windows. A *P*‐value <0.05 was determined to indicate statistical significance. BFMDRS and mFTMTRS scores were compared using the Wilcoxon signed rank test for paired variables. To justify the use of a non‐parametric test, the Kolmogorov–Smirnov test was used to assess variables for normal distribution.

## Results

Eighteen consecutive patients with dystonic head tremor were included in the present study, of whom 8 (44.45%) had Vim and GPi electrodes implanted and 10 (55.55%) had Vim DBS only. Operations took place over a period of 18 years. Patients were recruited over a period of 19 years. All patients had Vim stimulation activated after the trial period. One patient had GPi DBS stimulation additionally activated 1 year after the operation due to insufficient dystonia reduction.

For long‐term follow‐up the shortest available period was 2 years, and the longest was 16 years, with a mean long‐term follow‐up of 7.3 years. Overall, 17 patients completed follow‐up after a minimum of 2 years, 10 patients were evaluated for longer than 5 years after the operation, and in 5 patients follow‐up was available for more than 10 years.

Demographic and clinical data are summarized in Table 1. Ten patients were female (55.56%). Mean age at the time of surgery was 53.28 years. They had suffered from dystonia between 4 and 61 years (mean 22.22 years). In 17 patients, dystonia was classified as idiopathic, in 10 as sporadic (55.55%), and in 7 as familial (38.89%), whereas 1 patient had acquired dystonia (5.56%) after severe brain injury resulting in bifrontal lesions. One patient had a positive family history of essential tremor and 1 of head tremor.

**TABLE 1:** Demographic and clinical data of 18 patients with dystonic head tremor

Variable	Mean value/percentage	SD
Age (years)	53.28	14.03
Female/male	10 (55.56%)/8 (44.45%)	
Dystonia since age (years)	31.06	17.06
Dystonia duration at surgery (years)	22.22	15.63
Etiology of dystonia		
Idiopathic: sporadic	10 (55.55%)	
Idiopathic: familial	7 (38.89%)	
Acquired	1 (5.56%)	
Anti‐dystonic medication preoperatively	18 (100%)	
BTX in medical history	11 (61.11%)	
Anti‐dystonic medication postoperatively	6 (33.33%)	
Target		
Vim only	10 (55.55%)	
Vim + GPi	8 (44.45%)	

Abbreviations: SD, standard deviation; BTX, botulinumtoxin; Vim, nucleus ventralis intermedius; GPi, globus pallidus internus.

Dystonic head tremor was the most prominent symptom in all patients. Tremor was irregular and appeared with different amplitudes. Patients used sensory tricks to suppress the tremor, and cognitive or emotional stress aggravated the tremor. In addition to head tremor, 11 patients had mild tremor involving their arms, and in 4 patients the voice was affected. All patients had received medical treatment at some point before the operation, addressing tremor and/or dystonia. Medication included propranolol, primidone, anti‐cholinergic and dopaminergic drugs. Eleven patients (61.11%) had BTX injections. Six patients continued their medication after the operation (33.33%), but none continued BTX injections postoperatively. For a detailed characterization of the individual patients see Table 2.

**TABLE 2:** Individual patient data (N = 18)

Patient	Age at DBS	Dystonia since age	Etiology	Body distribution of dystonia	Tremor	BFMDRS‐Motor pre‐operatively	mFTM‐TRS pre‐operatively	Last available long‐term follow‐up			
								Months	≥ 2 y	≥ 5 y	≥ 10 y
No. 1	45	6	idiopathic: sporadic	neck, arms	head, arms	6	3	84	+	+	
No. 2	56	41	idiopathic: familial	neck, arms	head, arms	16	8	40	+		
No. 3	68	7	idiopathic: sporadic	neck, arms	head, arms, voice	22	6	50	+		
No. 4	65	40	idiopathic: sporadic	neck, arms	head, arms, voice	4	10	22			
No. 5	43	33	idiopathic: sporadic	neck, arms	head, arms	8	8	37	+		
No. 6	71	50	idiopathic: familial	neck, larynx	head, voice	7	10	62	+	+	
No. 7	63	9	idiopathic: familial	neck, larynx, arms	head, arms	20	5	120	+	+	+
No. 8	70	64	idiopathic: sporadic	neck, arms	head, arms	9,5	6	50	+		
No. 9	38	15	idiopathic: familial	neck, arms	head, arms	16	9	131	+	+	+
No. 10	43	20	idiopathic: sporadic	face, neck	head	1	10	64	+	+	
No. 11	54	42	idiopathic: sporadic	neck, arms	head, arms	14	6	33	+		
No. 12	50	20	idiopathic: familial	face, neck, arms	head, arm	22	10	100	+	+	
No. 13	43	33	idiopathic: sporadic	neck, arms	head	24	10	44	+		
No. 14	74	48	acquired: trauma	face, neck, trunk	head	21	12	215	+	+	+
No. 15	45	33	idiopathic: sporadic	face, neck	head	4	11	24	+		
No. 16	68	53	idiopathic: familial	neck, arms	head	21	5	112	+	+	
No. 17	33	29	idiopathic: familial	face, neck, larynx, arms	head, arms, voice	12	8	142	+	+	+
No. 18	30	16	idiopathic: sporadic	neck, arms, trunk	head	24	8	206	+	+	+

Abbreviations: BFMDRS‐M, Burke‐Fahn‐Marsden Dystonia Rating Scale motor score; mFTMTRS, modified Fahn‐Tolosa‐Marin Tremor Rating Scale; y, years; age given in years.

All postoperative stereotactic scans confirmed appropriate electrode placement in the target. No bleedings were found. During chronic stimulation 2 patients experienced dysarthria, and 2 patients showed mild gait ataxia. All side effects were resolved by re‐programming of the stimulation settings.

**FIG. 1:**
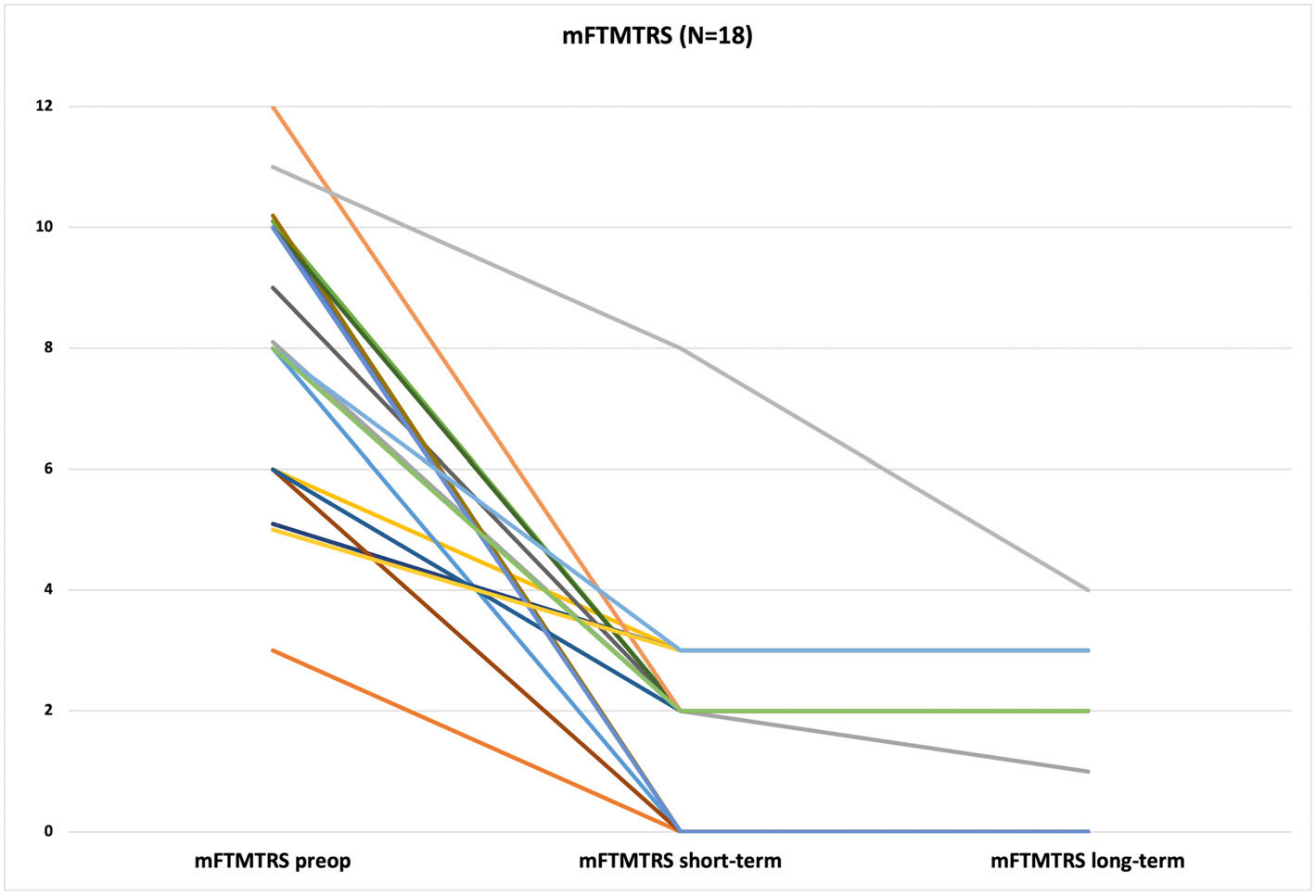
Modified Fahn‐Tolosa‐Marin Tremor Rating Scale (mFTMTRS) for individual patients before and after ventral intermediate deep brain stimulation (Vim DBS). Individual patient scores (n = 18) are marked by different colors.

The mean mFTMTRS preoperatively was 7.94 (±2.46) and decreased significantly at short term follow‐up to a score of 2.0 (±1.94) (*P* < 0.001), indicating a tremor reduction of 74.81% after a minimum of 3 months of stimulation. Tremor improvement was stable at long‐term follow‐up with a mean mFTMTRS of 1.71 (±1.31) (*P* < 0.001) after more than 2 years of chronic stimulation (see Table 2; Fig. 1).

**FIG. 2:**
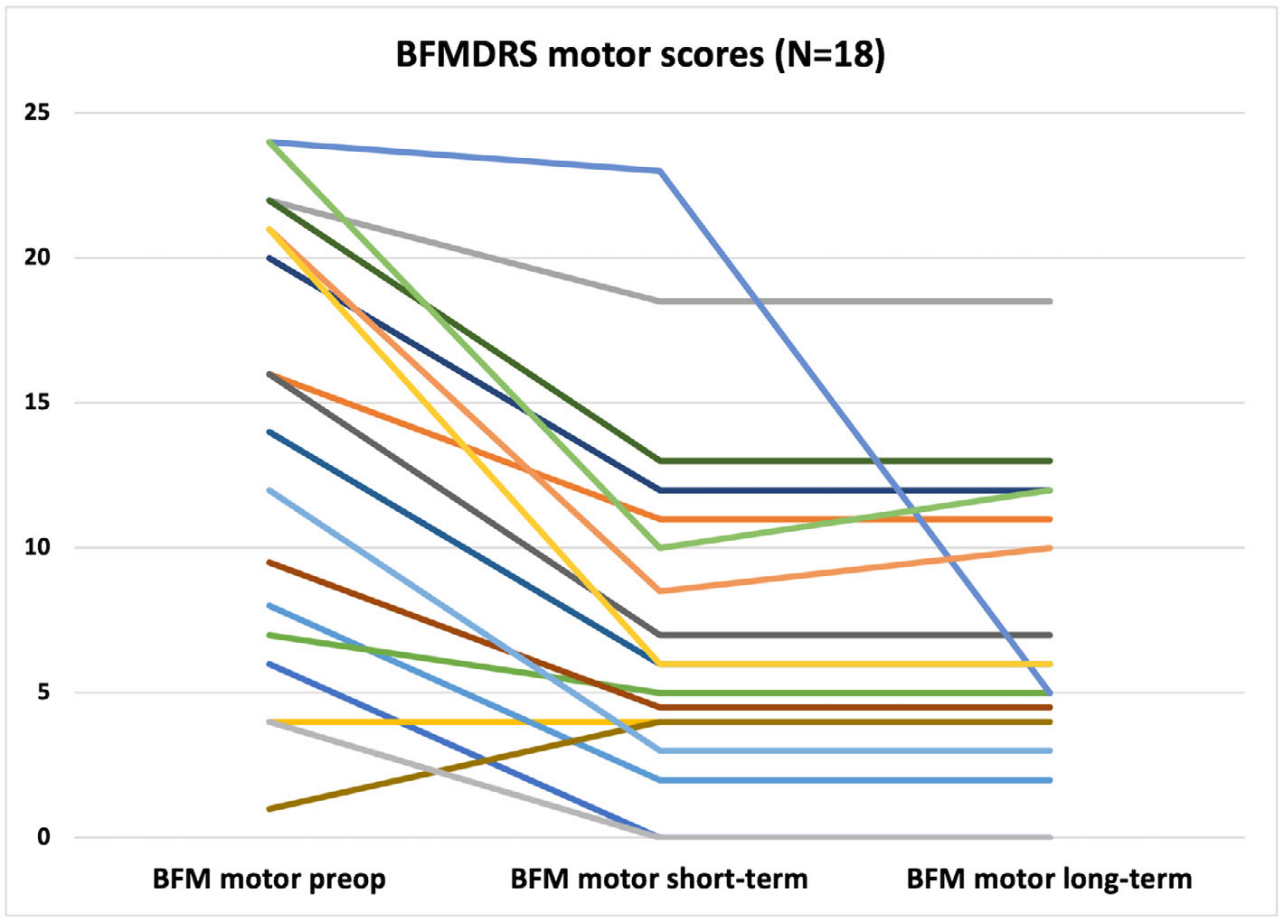
Burke‐Fahn‐Marsden Dystonia Rating Scale motor (BFMDRS) motor scores for individual patients before and after ventral intermediate deep brain stimulation (Vim DBS). BFMDRS motor scores for individual patients (n = 18) are marked by different color.

Under chronic Vim stimulation, dystonia also improved markedly from a mean BFMDRS‐M score of 14.56 (±7.53) to 7.85 (±6.25) at short‐term follow‐up (*P* < 0.001) and to a score of 7.0 (±5.05) at long‐term follow‐up (*P* < 0.001), indicating a stable response (see Table 2; Fig. 2). The same is reflected in the BFMDRS‐D, which improved from a mean score of 3.88 (±2.42) before the operation to 2.65 (±3.28) and 2.71 (±3.46) at short‐ and long‐term follow‐up, meeting also statistical significance (*p* < 0.05) (see Table 3). A representative case of clinical improvement with thalamic DBS in a 43‐year‐old patient with head tremor and cervical dystonia is captured in Video 1.

**TABLE 3:** Dystonia and tremor rating scale scores for 18 patients before and after Vim DBS

	Preop (N = 18)				Postop short** (N = 18) (3–24 months)				Postop long** (N = 17) (> 24 months)			
	Mean	SD	Median	IQR	Mean	SD	Median	IQR	Mean	SD	Median	IQR
BFMDRS‐M	14.56	7.53	15	15.9	7.85*	6.25	6	6.75	7.0*	5.05	6	7
BFMDRS‐D	3.88	2.42	4	4	2.65*	3.28	1,5	3.5	2.71*	3.46	1	4
mFTMTRS	7.94	2.46	8	4	2.0*	1.94	2	2.75	1.71*	1.31	2	3

*Note*: Kolmogorov–Smirnov Test: BFMDRS‐M preop: *P* = 0.164; BFMDRS‐M postop short: *P* = 0.200; BFMDRS‐M long: *P* = 0.200; BFMDRS‐D preop: *P* = 0.051; BFMDRS‐D postop short: *P* = 0.026; BFMDRS‐D long: *P* = 0.030; mFTMTRS preop: *P* = 0.200; mFTMTRS postop short: *P* = 0.008; mFTMTRS long: *P* = 0.013.

Abbreviations: SD, standard deviation; IQR, interquartile range; BFMDRS‐M, Burke‐Fahn‐Marsden Dystonia Rating Scale motor score; BFMDRS–D, Burke‐Fahn‐Marsden Dystonia Rating Scale disability score; mFTMTRS, modified Fahn‐Tolosa‐Marin Tremor Rating Scale.

* *P* < 0.05 in Wilcoxon signed‐rank test;

** *P* < 0.001 in Wilcoxon signed‐rank test.

**VIDEO 1:** Clinical course of a 43‐year‐old patient with dystonic head tremor before and after thalamic deep brain stimulation (DBS) surgery. Segment 1: Before surgery with head at rest, finger to nose test, drinking from a cup, writing and gait. Segment 2: After surgery at rest and drinking from a cup. The dystonia is more prominent when the head tremor is reduced. Segment 3: After 9 years of thalamic DBS; at rest, finger to nose test, drinking from a cup. Tremor is remitted, and dystonia is markedly improved in the long term.

One patient developed an infection at the IPG implantation site, which was successfully treated with antibiotics and surgery. Another patient developed an infection at the burr hole site, which was treated with antibiotics and plastic surgery. Due to continued problems with wound healing, however, the whole DBS system was removed 3 years after the implant procedure. As the patient had ongoing improvement in tremor as well as in dystonia after removal of the DBS system, he refrained from re‐implantation after full recovery from the infection.

DBS settings were programmed initially with bipolar stimulation of both middle contacts with a large pulse width (210 μs) and a frequency of 130 Hz. Stimulation settings were adjusted in all patients before discharge and regularly during follow‐up visits (every 3 to 6 months) in the first year postoperatively to improve the treatment effect or reduce stimulation‐induced side effects.

The stimulation settings for individual patients in the early postoperative period and at the last available follow‐up are shown in Tables 4 and 5. The mean stimulation voltage in the early postoperative period was 1.91 V for both the right and left hemispheres, ranging from 0.9 V to 3.8 V. Mean pulse width was 205 μs, and mean frequency 130.28 Hz. Three patients had monopolar stimulation. At the last available follow‐up, the mean voltage increased to 2.38 V for the right hemisphere and to 2.36 V for the left hemisphere. In 5 patients, pulse width was reduced over time (in 3 patients to 60 μs, and in 2 patients to 180 μs).

**TABLE 4:** Stimulation settings for individual patients in the early postoperative period

Patient	Early post‐op follow‐up	
	Right hemisphere	Left hemisphere
1	bipolar; 1−/2+; 0.8 V; 210 μs; 130 Hz	bipolar; 9−/10+; 0.8 V; 210 μs; 130 Hz
2	bipolar; 1−/2+; 1.2 V; 210 μs; 130 Hz	bipolar; 9−/10+; 1.2 V; 210 μs; 130 Hz
3	monopolar; 11−/C+; 2.5 V; 210 μs; 130 Hz	monopolar; 3−/C+; 2.5 V; 210 μs; 130 Hz
4	monopolar 1−/C+; 1.4 V; 210 μs; 130 Hz	monopolar 9−/C+; 1.4 V; 210 μs; 130 Hz
5	bipolar; 0−/3+; 1.0 V; 210 μs; 130 Hz	bipolar; 8−/11+; 1.5 V; 210 μs; 130 Hz
6	bipolar; 1−/2+; 1.5 V; 210 μs; 130 Hz	bipolar; 9−/10+; 1.5 V; 210 μs; 130 Hz
7	bipolar; 1−/3+; 3.8 V; 210 μs; 130 Hz	bipolar; 5−/7+; 3.3 V; 210 μs; 130 Hz
8	bipolar; 0−/1+; 1.0 V; 210 μs; 130 Hz	bipolar; 8−/9+; 1.0 V; 210 μs; 130 Hz
9	bipolar; 1−/2+; 2.5 V; 210 μs; 130 Hz	bipolar; 5−/6+; 2.5 V; 210 μs; 130 Hz
10	bipolar; 0−/2+; 2.0 V; 180 μs; 150 Hz	bipolar; 8−/10+; 1.9 V; 180 μs; 150 Hz
11	bipolar; 1−/2+; 1.5 V; 210 μs; 130 Hz	bipolar; 6−/7+; 1.5 V; 210 μs; 130 Hz
12	bipolar; 0−/1+; 1.5 V; 210 μs; 130 Hz	bipolar; 0−/1+; 1.5 V; 210 μs; 130 Hz
13	monopolar; 9−/C+; 1.6 V; 150 μs; 100 Hz	monopolar; 13−/C+; 1.6 V; 150 μs; 100 Hz
14	monopolar; 0−/C+; 2.7 V; 210 μs; 145 Hz	monopolar; 0−/C+; 2.7 V; 210 μs; 145 Hz
15	bipolar; 0−/1+; 2.0 V; 210 μs; 130 Hz	bipolar; 8−/9+; 2.0 V; 210 μs; 130 Hz
16	bipolar; 1−/2+; 3.0 V; 210 μs; 130 Hz	bipolar; 5−/6+; 3.0 V; 210 μs; 130 Hz
17	bipolar; 0−/1+; 3.6 V; 210 μs; 130 Hz	bipolar; 2−/3+; 3.6 V; 210 μs; 130 Hz
18	bipolar; 0−/3+; 0.9 V; 210 μs; 130 Hz	bipolar; 0−/3+; 0.9 V; 210 μs; 130 Hz

*Note*: Settings given per electrode: stimulation mode (monopolar/bipolar); amplitude (V); pulse width (μs); frequency (Hz); C = case.

**TABLE 5:** Stimulation settings for individual patients at the last available follow‐up

Patient	Last available follow‐up	
	Right hemisphere	Left hemisphere
1	bipolar; 1−/2+; 1.5 V; 210 μs; 130 Hz	bipolar; 9−/10+; 2.4 V; 210 μs; 130 Hz
2	bipolar; 5−/6+; 3.1 V; 210 μs; 130 Hz	bipolar; 9−/10+; 2.4 V; 210 μs; 130 Hz
3	monopolar; 11−/C+; 2.9 V; 210 μs; 130 Hz	monopolar; 3−/C+; 2.5 V; 210 μs; 130 Hz
4	monopolar 1−/C+; 2.4 V; 210 μs; 130 Hz	monopolar 9−/C+; 2.4 V; 210 μs; 130 Hz
5	bipolar; 0−/3+; 2.5 V; 210 μs; 130 Hz	bipolar; 8−/11+; 3.6 V; 210 μs; 130 Hz
6	bipolar; 0−/2+; 2.0 V; 210 μs; 130 Hz	bipolar; 8−/10+; 2.0 V; 210 μs; 130 Hz
7	bipolar; 9−/10+; 4.4 V; 210 μs; 130 Hz	bipolar; 13−/14+; 4.7 V; 210 μs; 130 Hz
8	monopolar; 0−/C+; 1.5 V; 210 μs; 130 Hz	monopolar; 8−/C+; 1.5 V; 210 μs; 130 Hz
9	bipolar; 1−/2+; 1.8 V; 210 μs; 130 Hz	bipolar; 5−/6+; 1.8 V; 210 μs; 130 Hz
10	bipolar; 0−/2+; 2.2 V; 180 μs; 150 Hz	bipolar; 8−/10+; 2.1 V; 180 μs; 150 Hz
11	bipolar; 1−/2+; 1.8 V; 210 μs; 130 Hz	bipolar; 5−/6+; 1.8 V; 210 μs; 130 Hz
12	bipolar; 8−/C+; 3.1 V; 180 μs; 130 Hz	bipolar; 0−/C+; 2,7 V; 180 μs; 130 Hz
13	monopolar; 8−/9−/C+; 1.0 V; 60 μs; 100 Hz	monopolar; 12−/13−/C+; 1.0 V; 60 μs; 100 Hz
14	monopolar; 0−/C+; 2.8 V; 210 μs; 145 Hz	monopolar; 0−/C+; 2.7 V; 210 μs; 135 Hz
15	monopolar; 1−/C+; 2.2 V; 180 μs; 180 Hz	bipolar; 8−/C+; 2.2 V; 180 μs; 180 Hz
16	monopolar; 0−/C+; 3.2 V; 60 μs; 150 Hz	bipolar; 4−/C+; 3.2 V; 60 μs; 150 Hz
17	bipolar; 0−/1+; 3.0 V; 240 μs; 130 Hz	bipolar; 2−/3+; 2.0 V; 240 μs; 130 Hz
18	monopolar; 1−/C+; 1.5 V; 60 μs; 130 Hz	monopolar; 0−/C+; 1.4 V; 210 μs; 130 Hz

*Note*: Settings given per electrode: stimulation mode (monopolar/bipolar); amplitude (V); pulse width (μs); frequency (Hz); C = case.

## Discussion

Our results show that bilateral DBS of the thalamic Vim is a safe and efficient treatment for dystonic head tremor in patients where head tremor is the primary symptom. Ten patients had stable improvement for more than 5 years, and in 5 patients thalamic DBS reduced tremor and dystonia for more than 10 years. In single instances improvement can be sustained up to 16 years. In addition to tremor, dystonia symptoms were markedly improved by Vim DBS in our cohort. However, dystonic posturing was only mild to moderate in this group of patients. Side effects were few and could be managed effectively, and in particular thalamic DBS did result neither in bradykinesia nor in dyskinesias as observed with DBS in other targets in dystonia.^12^, ^36^

Although pallidal DBS nowadays has become accepted as standard treatment for dystonia, it showed mixed results in patients with dystonic tremor.^8^, ^12^, ^18^, ^19^, ^22^, ^31^, ^37^ Variable improvement was seen in few patients with dystonic tremor of the extremities or of the head; amelioration of dystonia, however, was more marked than that of tremor.^12^, ^22^ The response of pallidal DBS for dystonic head tremor appears to be particularly difficult to predict. Single case reports also report complete failures.^14^, ^18^, ^19^, ^37^ The thalamus has been considered a target for treatment of dystonic tremor mainly with regard to the results of chronic DBS in the treatment of essential tremor and other types of tremor.^30^, ^38^ Although for some time tremor and dystonia were considered symptoms of dysfunctions in different neuronal circuits, overlap in these pathways involving thalamic structures have been recognized.^46^ Therefore, thalamic as well as pallidal stimulation seem to be reasonable approaches for treatment of dystonic head tremor. Recently, Paschen et al. compared GPi to Vim DBS in dystonic tremor and to Vim DBS in essential tremor and found the same efficacy for tremor reduction for all 3 groups. In the GPi group they reported 60% improvement in overall tremor as well as head tremor and 47% improvement in dystonia. They described a trend toward more dystonia improvement in pallidal DBS, but targets were chosen due to the dominant symptom, giving the GPi DBS group higher preoperative dystonia scores, which might have influenced results.^17^ Our case series showed 74.2% tremor improvement at short‐term follow‐up and 46.1% dystonia improvement at short‐term as well as 51.9% at long‐term follow‐up. Therefore, our results show more tremor improvement than with GPi DBS, whereas dystonia improvement seemed to be comparable to pallidal DBS, taking into account that numbers published previously varied between 47% and 60%.^17^, ^22^ However, we need to emphasize that comparisons between these studies can only give an estimate, because different rating scales were used throughout the literature. Furthermore, in most patients dystonic movements were less pronounced than tremor.

Several reports on thalamic DBS for dystonic tremor have been published thus far.^14^, ^18^, ^21^, ^22^, ^23^, ^24^, ^25^, ^31^, ^39^, ^40^, ^41^, ^42^ Improvement rates of tremor ranged between 29% up to 89% when combined with subthalamic area stimulation.^14^, ^16^, ^23^ Dystonia improved by 70.4% under Vim/subthalamic DBS and even up to 87.5% in single cases.^21^, ^23^ Shepherd et al. reported 33.9%–43.1% tremor reduction in dystonic tremor patients after 1 year of Vim/zona incerta stimulation. They found significantly better responses in essential tremor patients, but a habituation to DBS in both groups appeared in the long term.^15^ A systematic review by Tsuboi et al. showed that subscores for head tremor improved significantly greater in patients with bilateral DBS than in those with unilateral DBS.^43^

Less information has become available on the effect of thalamic DBS on patients presenting with dystonic head tremor as the leading symptom. According to two small case series and few case reports, outcome has been favorable, in general.^17^, ^21^, ^22^, ^23^, ^25^ A comparative analysis of the results from these reports, however, is difficult because different thalamic/subthalamic targets were used.^17^, ^21^, ^22^, ^23^, ^25^ Pauls and colleagues reported follow‐up results after 3 months of chronic thalamic/subthalamic DBS in 7 patients. Dystonic head tremor was improved by a mean of 57.1% according to a modified FTMTRS (as used in the present study), and dystonia by a mean of 70.4% according to the BFMDRS‐M.^21^ Yamahata et al. reported “complete control” of dystonic head tremor in a woman with combined thalamic Vim/nucleus ventro‐oralis anterior (Voa) stimulation over the course of 5 years.^25^

Remarkably, the thalamus was the main target for treatment of dystonia in the radiofrequency lesioning era.^27^, ^28^, ^29^ The majority of thalamotomy trials reported improvement of dystonic symptoms. However, with regard to the different concepts of classifying dystonia, the lack of appropriate rating scales, and variable follow‐up periods it is not possible to determine the degree of the improvement. There is little information available from these early reports on amelioration of dystonic tremor, and one must be aware that the concept of dystonic tremor during that period had not been discussed yet. Furthermore, several thalamic targets were used either alone or in combination.^27^, ^28^, ^29^, ^44^

In the present study we used the Vim (which corresponds to the ventralis lateralis posterior [VLp] nucleus in the Jones terminology) as target for the placement of the DBS electrodes. With regard to the somatotopy of the Vim we chose a target that was slightly more medial than that for DBS treatment of tremor of the extremities in the majority of cases. The Vim is the thalamic region that receives input from the cerebellum, and thus considering accumulating knowledge on dystonic network connectivity it might not only be involved in the pathophysiological circuitry of tremor but also in that of dystonia.^32^, ^45^, ^46^ Other thalamic regions of interest for DBS in dystonia that have also been applied during the thalamotomy era include more anteriorly located nuclei within the VL. These comprise (according to Hassler' s nomenclature) the nucleus ventro‐oralis posterior (Vop), which is now considered as an interface between the ventralis lateralis anterior (VLa) nucleus and the VLp, the Voa (corresponding to the anterior VLa), and the nucleus ventro‐oralis internus (Voi). The Voa/VLa is the major pallidal receiving area. In addition, the subthalamic region with its nuclei and fiber bundles has been and is being targeted.^15^, ^21^ Combined Vim/PSA proved effective in isolated head tremor and dystonic tremor of the limbs with complete tremor control in few patients.^16^

Although head tremor was the dominant symptom in the patients of our study, some component of dystonia was evident in all instances. In that regard, it needs to be pointed out that the terms *isolated head tremor* and *dystonic head tremor* have not been used in a consistent way. *Isolated head tremor* was originally classified as a subtype of essential tremor, whereas *dystonic tremor* has been defined as tremor that is manifest in a body region affected by dystonia.^2^ Thus, some patients with head tremor might either be classified as presenting with a manifestation of (essential) tremor or with a dystonic disorder. For example, if in addition to head tremor, action or postural tremor is present in the upper limbs, the disorder could be classified as an essential tremor syndrome.^3^ Ferrazzano et al. recently showed that 75% of patients with initially isolated head tremor developed a dystonic posture after 5 years.^47^ Therefore, isolated head tremor was also classified as a dystonic movement disorder recently.^48^ Taking these semantic difficulties into account, a precise and cautious interpretation of the available literature on the surgical treatment of head tremor is elementary to identify the most appropriate therapeutic options.^22^, ^49^

Although tremor and dystonia rating scale scores both were ameliorated with thalamic DBS in patients with dystonic tremor, in most cases more improvement was seen for tremor (as in our study) than for dystonia.^16^, ^21^, ^31^ Paschen et al. reported no significant difference in dystonia improvement; however, they found a trend toward less dystonia improvement with Vim DBS (3.8 vs. 1.2 points reduction in UDRS).^17^ Overall we report more improvement in dystonia compared to other trials with thalamic DBS. Reasons for this might be that patients were affected more or less severely by dystonia, and a variability in thus far unknown genetic backgrounds that could respond more or less to chronic DBS. However, the choice of DBS electrode positioning, including Voa or Vop, and the angle between the entry point of the electrode with regard to the target, might also be relevant.^21^

Combined approaches targeting both thalamic and pallidal targets provide an option to overcome the manifold uncertainties about the choice which structure should be targeted. We have used this strategy in the first 8 patients of our series, but abandoned it thereafter because in all cases tremor was more improved with thalamic than with pallidal stimulation during the trial period. However, we cannot exclude a lesioning effect through the pallidal electrodes, especially taking into account the 1 patient who showed continued response after electrode removal. Chronic stimulation of 2 targets in patients with dystonic tremor has been reported in a few patients over the past 2 decades providing partially inconsistent results.^20^, ^22^, ^24^, ^31^ Only recently, such a double‐target strategy has been studied in a larger group of patients in a more systematic way as a treatment option for dystonic tremor.^22^ Paoli and colleagues reported a series of 9 patients with dystonic tremor with chronic stimulation of both Vim and GPi who had an improvement of dystonia of 75.8% according to the BFMDRS‐M along with amelioration of tremor at 1‐year follow‐up. Unfortunately, a specific tremor rating scale before and after surgery had not been applied.^22^ Head tremor was present in the majority of these patients, but in none of them it was the leading symptom. These results are comparable to Yilmaz et al., who used combined Vim/PSA DBS without pallidal stimulation.^16^

It is unclear which stimulation settings would yield the best results for dystonic head tremor with thalamic stimulation. A wide variety of different stimulation settings have been reported in cases with thalamic stimulation for dystonia and dystonic tremor regarding the mode of stimulation (monopolar vs. bipolar), amplitude, pulse width, and frequency.^14^, ^21^, ^30^ We here used stimulation settings that we apply also as standard for Vim stimulation in essential tremor. In many patients a relatively low amplitude of the voltage was sufficient to control tremor in the early postoperative period, possibly secondary to a lesioning effect. The amplitude of stimulation voltage increased in the long term to control tremor in most instances; however, we did not encounter cases with marked loss of stimulation effect. The reason that the frequency of gait ataxia and speech disturbances was relatively low and could be managed in all cases might be that the thalamic target for electrode implantation was more medial than that used for tremor of the extremities in essential tremor.

Although our study on this topic was based on a meticulous patient selection and application of standard rating scales, it has several limitations. First of all, follow‐up data were collected in a retrospective fashion, and postoperative videotaping was not available in all instances for the different follow‐up periods. Further, surgeries were performed over a long period of 16 years that might have had an impact on treatment routines. In addition, the limited patient number resulted in the use of nonparametric testing that achieves robustness by sacrificing power of the analysis; therefore, only larger effects can be detected. Also, genetic testing was not widely available at the time when the first patients were operated, and it is still not routinely performed in patients with dystonic head tremor when no other neurological symptoms are present. Therefore, we cannot scrutinize for differences in outcome with regard to different genetic backgrounds. Furthermore, as this was an observational study with retrospective analysis of data, and all patients with dystonic head tremor received thalamic DBS, we cannot provide a direct comparison between pallidal and thalamic DBS. Although this study provides favorable results for thalamic DBS in dystonic head tremor patients, we cannot provide sufficient data for arm tremor improvement, although 11 patients had tremor and dystonia of the upper extremities in our cohort. As we have used the mFTMTRS to focus on head tremor, we did not systematically investigate arm tremor. Arm tremor was less pronounced than head tremor in all cases. Previously, Aasef et al. have pointed out the difference between tremor associated with dystonia (where tremor and dystonia develop in different body regions) and dystonic tremor, where tremor develops in the same body part that is affected by dystonia.^50^ As there is no guideline on what DBS target is more beneficial for these patients, it would be interesting to perform a comparative analysis on these two tremor manifestations in dystonic patients.

Our results need to be confirmed by larger prospective randomized controlled studies. Due to the rarity of severe dystonic head tremor, such studies might be best performed within the frame of a multicenter design study to allow data collection over a reasonable period of time. For now we suggest considering thalamic Vim stimulation as a treatment option for predominant dystonic head tremor and mild to moderate dystonic symptoms.

## Author Roles

1. Research project: A. Conception, B. Organization, C. Execution. 2. Statistical analysis: A. Design, B. Execution, C. Review and critique. 3. Manuscript preparation: A. Writing of the first draft, B. Review and critique.

J.M.N.: 1C, 2A, 2B, 3A

M.E.W.: 1A, 1B, 1C, 2A, 3B

C.B.: 1A, 1B, 3B

J.R.: 1C, 2B, 3B

C.S.: 1C, 2B, 3B

D.D.: 1A, 2B, 3B

A.S.: 1B, 1C, 3B

J.K.K.: 1A, 1B, 1C, 2A, 2C, 3B

## Disclosures

**Ethical Compliance Statement**: We confirm that we have read the journal's position on issues involved in ethical publication and affirm that this work is consistent with those guidelines. The study was conducted in accordance with the Declaration of Helsinki, and all patients provided written informed consent to the use of anonymized patient data for research purposes. For the present retrospective analysis the Ethical Committee of the Hanover Medical School indicated that formal approval was not required.

**Funding Sources and Conflict of Interest**: No specific funding was received for this work. The authors declare that there are no conflicts of interest relevant to this work.

**Financial Disclosures for the Previous 12 Months**: J.K.K. has received consultant fees from Medtronic, as well as consultant fees from Boston Scientific, Inomed, and Aleva. J.M.N. has recieved travel grants from Boston Scientific. All other authors declare that there are no additional disclosures to report.

## Data Availability

The data that support the findings of this study are available on request from the corresponding author. The data are not publicly available due to privacy or ethical restrictions.
